# Infective Endocarditis in an Intravenous Drug User Leading to Myocardial Rupture and Hemopericardium

**DOI:** 10.1177/19253621231214442

**Published:** 2023-12-19

**Authors:** Cathy Cao, Jayantha Herath

**Keywords:** Forensic pathology, Infective endocarditis, Hemopericardium, Myocardial rupture, Intravenous drug use, Recurrence

## Abstract

**Introduction:**

Infective endocarditis (IE) is an infectious disorder of the innermost lining of the heart that can be fatal if left untreated. Infective endocarditis can spread beyond the endocardium into the myocardium and cause arrhythmias and myocardial wall rupture. Individuals with a history of intravenous drug use are at increased risk of developing IE and are at higher risk of dying, given their limited access to health care and adherence to treatment.

**Methods:**

A medicolegal autopsy was performed on a 30-year-old woman with a history of intravenous drug use and recent assault after a hospital admission during which she did not survive resuscitation.

**Results:**

The cause of death was found to be myocardial rupture in the setting of transmural IE. Postmortem imaging showed hemopericardium which was identified grossly with valvular vegetations in the heart. A ventricular wall defect along with transmural abscess formation was identified. Perimortem toxicology was positive for fentanyl, methamphetamine, and benzoylecgonine, a metabolite of cocaine. Postmortem blood cultures were positive for coagulase-negative *Staphylococci, Staphylococcus aureus, Candida tropicalis,* and *Viridians* group *Streptococci.* Postmortem tissue cultures taken from the heart was positive for *Candida glabrata* and *Streptococcus mitis*.

**Discussion:**

The decedent had significant risk factors for IE, such as intravenous drug use and a prior admission to hospital for IE. The organisms identified on culture are in-keeping with the gross findings of IE and the terminal event of myocardial rupture was likely the result of tissue damage resulting from IE.

## Introduction

Infective endocarditis (IE) is characterized by inflammation of the innermost layer of the heart, which can involve the cardiac valves, myocardium, chordae tendineae, and coronary arteries (1). Individuals with a history of immunosuppression, intravenous drug use, hemodialysis, and artificial heart valves are at increased risk of developing IE (2, 3). Valvular vegetations, abscesses, and associated pericarditis can be identified grossly on postmortem examination of IE cases (4) and other adverse complications include arrhythmias, reinfection, valve insufficiency, and structural destruction of heart structures (5). Microscopically, neutrophilic and inflammatory infiltrates can be seen in the endocardium and myocardium, and bacteria can be seen on Gram or Giemsa staining.

Postmortem blood and tissue cultures can be taken to determine the causative infectious agent; however, there is an increased risk of contamination compared to perimortem blood. *Staphylococcus aureus* is responsible for 20% to 30% of IE cases and is the bacterium reported in most cases of intravenous drug use-associated IE (4). Other bacterial agents of IE include streptococcal organisms contributing to another 30% of cases as well as enterococcal and gram-negative species in the HACEK group such as *Haemophilus, Aggregatibacter, Cardiobacterium, and Eikenella* species that are known primary causes of IE (1, 6, 7). Uncommonly, fungi such as species of *Candida* can also be responsible, as well as fastidious organisms such as *Coxiella burnetii, Tropheryma whipplei,* and *Bartonella* species which may not be identified on blood cultures (6, 7).

Specifically, in individuals with a history of intravenous drug use, there is an association with negative social determinants of health, access to health care and adherence to treatment. There is a higher rate of antimicrobial-resistant organisms in cases of IE in this demographic such as methicillin-resistant *Staphylococcus aureus*, as well as higher rates of IE recurrence and associated morbidity (8). There is also a higher incidence of complications during treatment for IE such as pulmonary emboli, increased size of valvular vegetations, and sepsis (3, 8). Outpatient therapeutic options are limited, as there is a risk of inappropriate use of central lines and peripherally inserted central catheters (PICC), and oral antibiotics alone are contraindicated in complicated or prolonged cases of IE (9).

In the current literature, there has not yet been a reported case of myocardial rupture in the setting of IE discovered on autopsy. Here, we present a case of ventricular wall rupture and hemopericardium secondary to recurrent IE resulting in the death of a 30-year-old female with a history of intravenous drug use.

## Case Report

A 30-year-old woman with a history of intravenous drug use was brought to the emergency department by bystanders who reported that she was confused. She had previously been hospitalized for IE five months prior and had left against medical advice during that admission. During this presentation to the hospital, she was described to have been hypotensive and sepsis was suspected, however, perimortem blood cultures were not taken. Fluids and antibiotics were administered, however, during an imaging study the following morning, she suddenly became short of breath and suffered a cardiac arrest and died when attempts to resuscitate her were unsuccessful. During her brief time in the hospital, the patient disclosed to medical staff that she had been assaulted the night before, and thus police were contacted upon her death.

Toxicologic analysis of perimortem blood taken in the hospital during the decedent’s admission was positive for fentanyl, methamphetamine, and benzoylecgonine, a metabolite of cocaine (Table 1), which is suggestive of intravenous drug use prior to the presentation described above. No fentanyl was reported to have been administered during the decedent’s course in hospital. The death certificate was completed by the coroner as “perforated bacterial endocarditis of the aortic valve in a woman with fentanyl, methamphetamine, and cocaine toxicity.”

**Table 1:** Results of toxicologic analysis of perimortem blood taken in the hospital at the start of admission.

**Substance in** hospital bl**ood**	**Result**
**Fentanyl**	11 ng/mL
**Methamphetamine**	0.34 mg/L
**Amphetamine**	0.083 mg/L
**Benzoylecgonine**	0.30 mg/L
**Cocaine**	Not detected
**Ethanol**	Not detected

**Figure 1:**
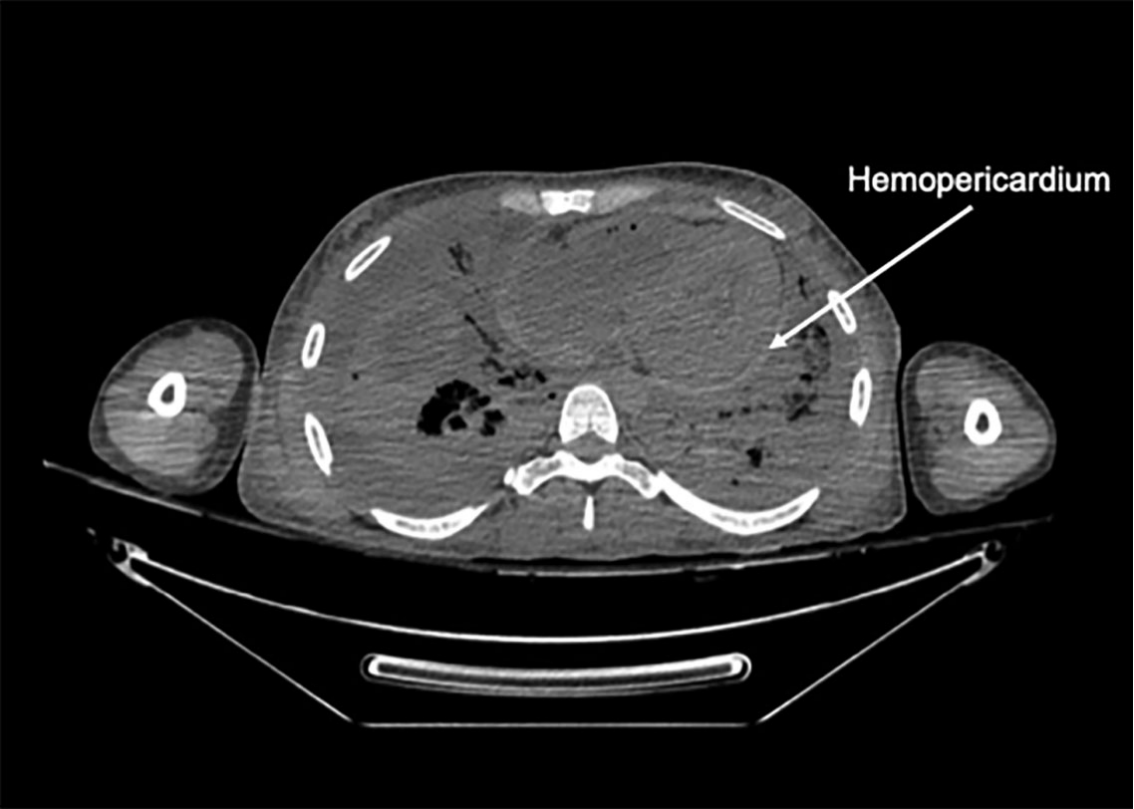
Postmortem computed tomography (CT) scan of the decedent, revealing fluid in the pericardial space, in-keeping with hemopericardium identified on internal examination.

**Figure 2:**
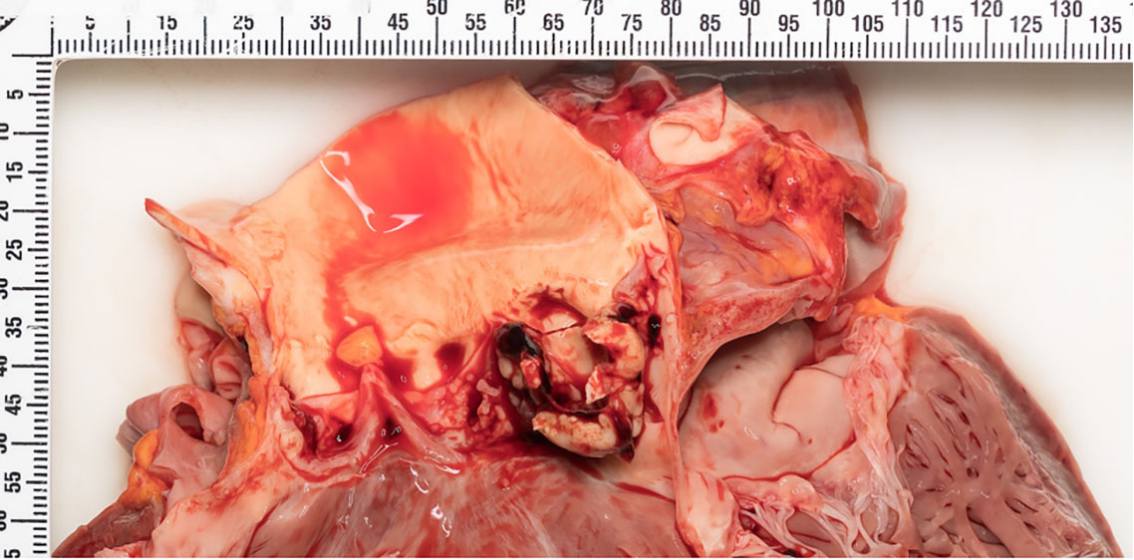
Extensive exophytic vegetations on the aortic valve are present, in-keeping with the clinical presentation of IE.

A complete medicolegal autopsy was performed, given the history of assault. The external examination demonstrated an adult female with no evidence of trauma or external injury. Postmortem computed tomography (CT) scan showed hemopericardium. Internal examination revealed hemopericardium as seen on the CT scan (Figure 1), chronic hepatitis, and fluid within the peritoneum and chest. The volume of hemopericardium was estimated to be approximately 160 mL of partially clotted blood. No other gross findings indicative of long-term drug use were identified. Further examination of the heart showed bacterial endocarditis with extensive vegetations on the aortic valve (Figure 2) and all three layers of the left ventricular wall were involved by abscess formation and a perforation into the pericardium was identified.

**Figure 3:**
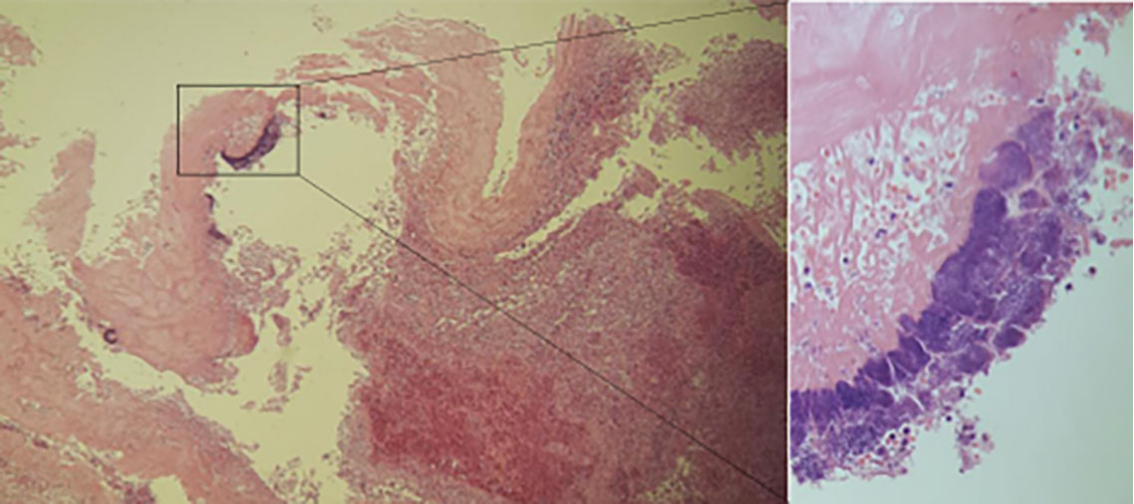
Bacterial colonies were identified on histology of the aortic valve, as seen on hematoxylin and eosin staining.

**Figure 4:**
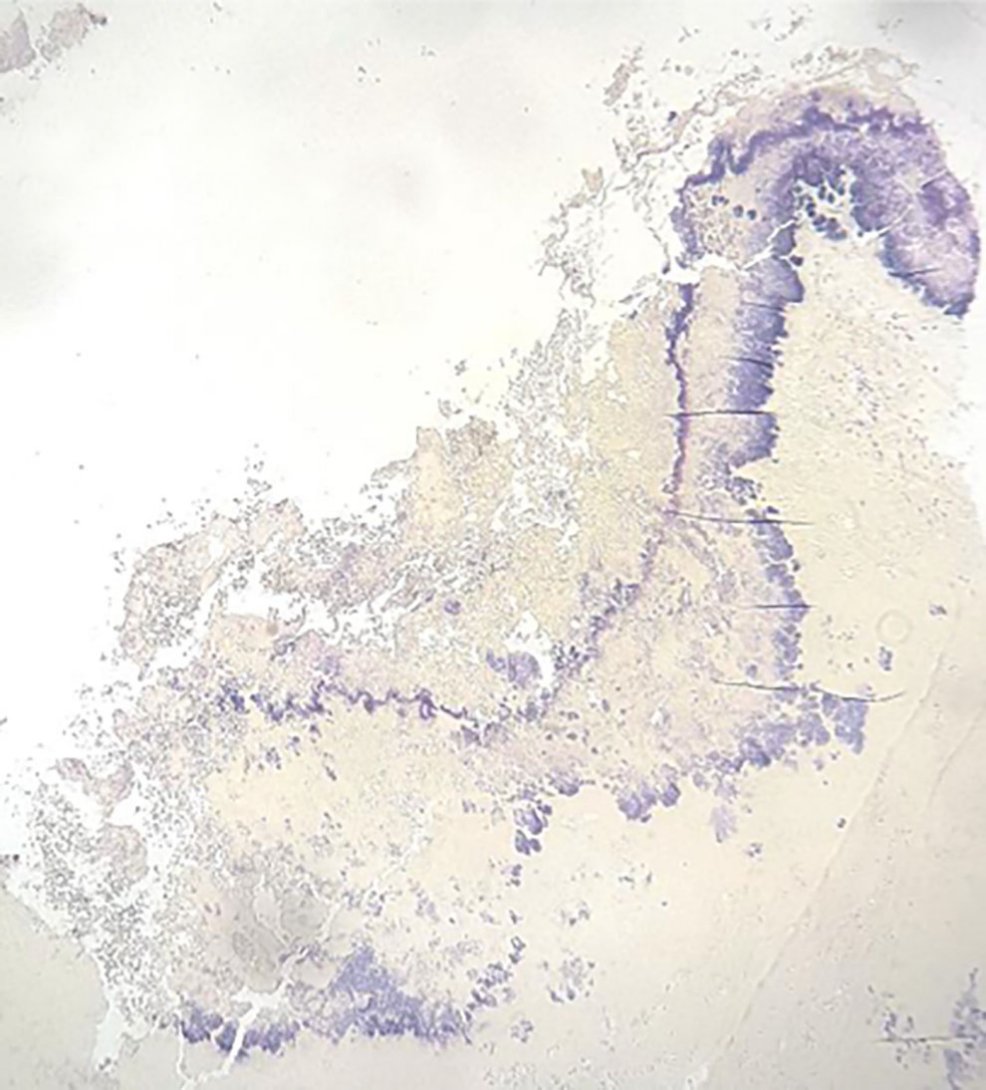
Gram-positive cocci identified on Gram staining of the aortic valve sections.

Postmortem microscopic analysis of the decedent’s heart showed signs of acute inflammation such as fibrin admixed with neutrophils as well as bacterial colonies on the aortic valve (Figure 3). Gram-positive cocci were also identified on Gram staining (Figure 4). Postmortem cultures of the decedent’s blood was positive for coagulase-negative *Staphylococcus*, *Staphylococcus aureus, Candida tropicalis,* and *Viridians*-group *Streptococcus* (Table 2). Perimortem blood cultures were not taken in hospital, given the brevity of her course. Scant isolates of *Candida glabrata* (currently known as *Nakaseomyces glabratus*) and moderate isolates of *Streptococcus mitis* group organisms were detected from postmortem tissue cultures of the decedent’s heart (Table 3).

**Table 2:** Results from postmortem blood culture isolates. *Coagulase negative staphylococci may be contaminants, and clinical correlation is needed to interpret this result. This is in-keeping with the diagnosis of IE.

**1**	**Coagulase negative *Staphylococci* (not *S. lugdunensis*)**
**2**	*Staphylococcus aureus*
**3**	*Candida tropicalis*
**4**	*Viridians* group *Streptococci*

**Table 3:** Results from postmortem heart cultures taken from the aortic valve.

**Test**	**Result**
**Fungal isolate identification**	*Candida glabrata*
**Forensic** a**erobic** c**ulture**	*Streptococcus mitis* group, not *Streptococcus pneumoniae*

## Discussion

Infective endocarditis can have severe consequences such as sepsis, tissue destruction, and progressive heart failure. A validated risk score can be calculated to predict the mortality risk at 6 months, considering host factors, disease factors, and complications (10). The risk of complications is higher in individuals with comorbidities and lifestyle factors (3, 8, 11), such as the history of intravenous drug use described in the decedent above. While valvular vegetations are often described in association with IE (1, 3-5, 8, 11), other parts of the heart can be affected such as the coronary arteries, myocardium, chordae tendineae, and pericardium (4).

In the case of the above decedent, the integrity of the myocardium was likely compromised by the infection, given the proximity of the rupture site with an abscess, resulting in hemopericardium. The volume of hemopericardium in the setting of death after resuscitation may be confounded by chest compressions and other interventions, however, given there being limited space in the pericardial space, and even small volumes of hemopericardium can cause cardiac tamponade and reduce diastolic filling and stroke volume (12). The volume of hemopericardium required to cause issues in an individual is dependent on factors such as age, size of the individual’s heart, and if there are cardiac changes such as hypertrophic cardiomyopathy, further restricting the amount of blood the pericardium can accommodate. While extensive valvular vegetations were identified, these likely were not the immediate cause of death. It is unclear if the decedent had been experiencing complications from her prior presentation of IE, as she had signed out against medical advice likely before completing treatment. Untreated or incompletely treated, the risk of complications from IE is even greater (8,10).

The most contributory factor to the decedent’s presentation of IE was the history of intravenous drug use. The risk of nosocomial IE is higher in these individuals given their limited access to safe, clean paraphernalia and locations in which to use. Needle sharing is a known practice among users to conserve resources which increases the risk of bloodborne pathogens such as HIV and hepatitis. With the advent of safe injection sites and provision of clean needles, there has been a decrease in the overall risk of infections and thus long-term effects from infections are less frequently identified on postmortem exam.

The effects of some intravenous drugs, such as methamphetamine or cocaine can increase the risk of additional cardiac complications such as myocardial infarction and arrhythmias (13, 14). In our case, fentanyl, methamphetamine, and cocaine use were detected in the decedent’s perimortem blood, and the use of these substances further increased the decedent’s risk of myocardial rupture given the concurrent abscess formation affecting all three layers of the myocardium. Although there was no known history of cardiac event in the decedent, myocardial wall rupture can be a late complication of myocardial infarction. This would have been a more likely explanation of the myocardial rupture if findings of IE were absent.

Outpatient treatment options are limited for IE in the community and are difficult for these individuals to access, given socioeconomic factors limiting their ability for follow-up and finance medications in the absence of drug benefits. The first line of treatment for IE is intravenous antibiotics; however, this often requires a hospital admission where the patient can be observed. Oral antibiotics alone are not indicated as a first-line therapy in the treatment of IE, although they can be used as a step-down option to expedite recovery and prevent complications associated with prolonged intravenous antibiotic therapy (3, 7). Indwelling central lines or PICC lines are at risk of abuse, and pose an additional risk of infection, making outpatient intravenous therapy difficult. There is also an increased risk of these individuals developing antimicrobial-resistant IE, with inconsistent or incomplete antimicrobial treatment (1, 3), making IE increasingly difficult to treat in this demographic.

Clinically, the ability to effectively treat IE is largely dependent on the identification of the causative organism(s) on blood culture (3, 6-8, 15). Empiric broad-spectrum antibiotics are given initially, but the course is modified based on the result of blood culture, which may take several hours. To identify causative organisms during a postmortem examination in the absence of perimortem blood cultures, postmortem blood cultures can be performed at time of autopsy, however, the results of which can be limited given the higher likelihood of contamination and postmortem bacterial transmigration (16-18). In the case of the decedent described above, perimortem blood cultures were not performed, given the acuity of the decedent’s presentation. Autopsy was performed on the basis of assault allegations and sepsis was likely suspected after the decedent’s passing. In the postmortem blood culture, coagulase-negative *Staphylococcus, Staphylococcus aureus, Candida tropicalis,* and *Viridians* group *Streptococci* were identified (Table 2). *Staphylococcus aureus* is a known cause of IE in the setting of intravenous drug use (4); however, *Viridians* group *Streptococci* and coagulase-negative *Staphylocci* are known to cause IE in the setting of prosthetic heart valves (19, 20). While all of the organisms identified on postmortem culture are also known to be components of normal skin or mucosal flora, the results are in-keeping with a diagnosis of IE and it is difficult to determine the extent to which the results are affected by postmortem contamination.

Postmortem tissue culture may also be performed, and sterile technique is recommended to avoid introduction of contaminants from elsewhere in the body. The postmortem interval can influence culture results and is additionally influenced by factors such as perimortem hospital stay, tissue procurement technique, and the extent of decomposition of the body prior to being brought in for autopsy if the decedent died outside of hospital. Overall, the results of perimortem cultures were found to have a greater than 50% chance of being associated with a true pathologic agent (17). In our case, *Candida glabrata* and *Streptococcus mitis* were identified from postmortem cultures of the aortic valve. While rare, *Candida glabrata* has been reported as a cause of IE (21) and *Streptococcus mitis* belongs to the *Viridians* group *Streptococci* as identified above in the postmortem blood cultures. As above, these organisms are components of normal skin flora and mucosal flora respectively, and given the decedent’s history, *Staphylococcus aureus* is most likely the causative organism.

## Conclusion

In summary, IE can be associated with high mortality and morbidity, the risk of which are elevated in individuals with a history of intravenous drug use. This case report highlights a fatal complication of IE in which the myocardium may rupture due to extensive tissue destruction associated with the infectious process. While a medicolegal autopsy was performed in this case given the history of assault, a complete medical autopsy is warranted in similar cases, with careful examination of the heart to determine the extent of tissue damage if IE is suspected.

### Key Points

1. Intravenous drug use is associated with higher risk of developing IE.
2. IE can be difficult to treat, and the risk of reinfection is higher with therapeutic noncompliance, as is seen in many individuals who use intravenous drugs and individuals with inequitable access to health care.
3. Untreated IE can affect other heart structures such as the myocardium, leading to arrhythmias, heart failure, and myocardial rupture.
4. Infective endocarditis must be distinguished from other cardiac causes of sudden death in individuals with a history of intravenous drug use, such as myocardial infarction in the absence of IE.
5. The limitations of postmortem assessment of endocarditis include higher risk of contamination of blood and tissue culture, thus perimortem blood cultures are important in identifying the true causative agent.
